# Evaluating quality of palliative care from the perspective of healthcare professionals in different care settings: development of the quality of palliative care questionnaire – staff

**DOI:** 10.1186/s12904-026-02084-2

**Published:** 2026-04-10

**Authors:** Carina Werkander Harstäde, Anette Alvariza, Ulrika Östlund, Eva Benzein, Staffan Lundström, Marika Wenemark, Kristofer Årestedt

**Affiliations:** 1 Department of Health and Caring Sciences, Faculty of Health and Life Sciences, Linnaeus University, Kalmar/Växjö, Sweden; 2 Department of Health Care Sciences, Palliative Research Centre, Marie Cederschiöld University, Stockholm, Sweden; 3 Research and Development Department/Palliative Care, Stockholms Sjukhem, Stockholm, Sweden; 4 Centre for Research and Development, Uppsala University/Region Gävleborg, Gävle, Sweden; 5 Department of Public Health and Caring Sciences, Uppsala University, Uppsala, Sweden; 6 Faculty of Medicine and Health, School of Health Sciences, Örebro University, Örebro, Sweden; 7 Center for Collaborative Palliative Care, Linnaeus University, Växjö, Sweden; 8 Research and Development Department, Stockholms Sjukhem Foundation, Stockholm, Sweden; 9 Department of Oncology-Pathology, Karolinska Institute, Stockholm, Sweden; 10 Department of Health, Medicine and Caring Sciences, Linköping University, Linköping, Sweden; 11 Unit of Public Health and Statistics, Region Östergötland, Linköping, Sweden; 12 Department of Research, Region Kalmar County, Kalmar, Sweden

**Keywords:** Content validity, Healthcare professionals, Instrument development, Palliative care, Quality of care, Questionnaire

## Abstract

**Background and aim:**

Palliative care is provided at different care settings by different professionals, making management and organisation of services crucial. A comprehensive tool to systematically evaluate the quality of palliative care and identify potential areas for organizational improvement is highly warranted. The aim was to develop and examine the content validity of a questionnaire evaluating quality of palliative care from the perspectives of healthcare professionals in different care settings: the Quality of Palliative Care Questionnaire – Staff (QPCQ-S).

**Methods:**

This methodological study included booth qualitative and quantitative methods. The QPCQ-S was developed from existing questionnaires. Questions were modified to represent broad perspectives of palliative care before being reviewed by an expert group consisting of researchers in palliative care. A preliminary version was reviewed by healthcare professionals (*n* = 16), and the response options were examined, based on responses from 98 healthcare professionals who had participated in a development project. Cognitive interviews (*n* = 13) were performed with healthcare professionals. Finally, the questionnaire was evaluated by 421 healthcare professionals within a nursing home context.

**Results:**

Convergent validity was supported by the expert and advisory groups, and cognitive interviews. Problems with ceiling and floor effects was demonstrated for some items, which resulted in a more differentiated response scale for these items. A majority of the respondents reported the questions as important or fairly important (99.3%), and that the questions had provided them with the opportunity to give a true picture of palliative care (78.9%). However, a majority reported that the questions were difficult or fairly difficult to answer (64.0%), significantly more among nurse assistants compared to nurses, physicians and other professional with academic education (*p* = 0.031).

**Conclusion:**

The QPCQ-S showed good content validity, and the questions were reported important, giving a true picture of palliative care. Careful preparation is needed considering by whom and in what context the CPCQ-S are best used for evaluation of palliative care.

**Supplementary Information:**

The online version contains supplementary material available at 10.1186/s12904-026-02084-2.

## Background

Palliative care is characterized by a holistic approach, including both patients and their family members. It consists of preventing and relieving health related suffering of adults, children and their families when they are facing problems associated with life-threatening illness. The care is based on a comprehensive person-centred approach that addresses physical, psychological, social and spiritual suffering. This care aims to support people to live as fully as possible until they die and can be applicable from early to late in the course of illness [1, 2]. Today, palliative care is provided at different care settings by several different professionals, making the management and organisation of services crucial [3]. Therefore, it is valuable to capture perceptions of how this care is delivered from the perspective of healthcare professionals in different care settings as a way to evaluate its quality.

The concept of quality is complex and there are several definitions. Most of them suggest that quality can be seen as an expression concerning to what extent determined goals, demands or guidelines are fulfilled [4]. When evaluating care, different aspects such as structure, process and outcomes need to be considered. Structure refers to organizational factors defining the health system (physical characteristics such as resources, organisation of resources and management, and staff characteristics). Process refers to the interactions between users and healthcare structure (technical interventions and inter-personal interactions). Outcomes, finally, are the consequences of care, and here both structure and process have influence [5].

According to a recent worldwide survey [6], Sweden is one of the countries in the world delivering high standard palliative care at an advanced stage of integration to mainstream healthcare services. There has been a development of palliative care since the early 1980s and several initiatives have contributed to the high standard of care provided today. In 2013, the Swedish National Board of Health and Welfare (NBHW) published ‘National knowledge base guidance for good palliative care in end-of-life’ [7]. Furthermore, Sweden has a national care program in palliative care that was first established in 2013 and then revised in 2016 and 2021 [8]. In comparison with national policies in other countries, e.g., Australia and Great Britain, there are great resemblances concerning thoughts of dignity, symptom control, and person-centred care [9, 10], where the ultimate goal is to improve the quality of and access to palliative care. The Swedish guidelines also strive to create a common ground for palliative care in that they can be implemented in all kinds of care settings.

To evaluate palliative care, a national quality register in end-of-life care, the Swedish Register of Palliative Care (SRPC), was developed from principles for end-of-life care presented by the British Geriatrics Society [11, 12]. The register is based on retrospective questions focusing on quality of care during the patients’ last week of life assessed by healthcare professionals [13]. Data from the register show that palliative care has improved over the years since registration started in 2006 [14]. For example, the prevalence of six examined symptoms has decreased as well as prescription of ‘as needed’ medications for pain, nausea, anxiety and death rattle. Patients’ next of kin are more often offered a follow-up appointment after patients’ death. However, there is still potential for further progression. This is especially true for general palliative care [14]. Thus, continuous evaluations are required.

Despite the fact that the SRPC contributes important information about quality of care in end of life, it mainly reflects the palliative care during the last week of life. Palliative care needs are relevant under a much longer time than just this last week of life. Therefore, a comprehensive tool that can be used to systematically identify potential areas for organizational improvement to promote quality during the whole palliative period, would be of value. The tool should preferably cover all types of palliative care settings, any time during the period of palliative care, and different patient groups. To the best of our knowledge, this type of tool is lacking.

## Aim

The aim of this study was to develop and examine the content validity of a questionnaire evaluating quality of palliative care from the perspectives of healthcare professionals in different care settings: the Quality of Palliative Care Questionnaire – Staff (QPCQ-S).

## Methods and results

This methodological study, incorporating both qualitative and quantitative methods, was conducted to develop and evaluate the QPCQ-S. The study was carried out in two stages. Stage 1 included the development of the QPCQ-S, based on expert and advisory groups, cognitive interviews, and questionnaire data from a development project. Stage 2 included an overall evaluation of the instrument, based on data from an intervention study. According to the Swedish ethical review act (2003:460) [15], no ethical approval was required for the development stage of the QPCQ-S. For stage 2, the educational intervention study [16] had Ethical approval obtained from the Regional Ethical Review Board in Lund in Sweden (No. 2015/4). This study was conducted between 2015 and 2017.

### Stage 1: development of the QPCQ-S

#### Construction of questions and response options

The development of the QPCQ-S was guided by general recommendations for instrument construction [4] and performed by the authors who have extensive knowledge of palliative care, quality of care, healthcare organisation, psychometrics, and questionnaire design. In addition, National practice guidelines for palliative care [8] were considered. Questions were generated from already existing validated and in Sweden well established and frequently used questionnaires: Two questionnaires that is used to collect data for the Swedish Register of Palliative Care (SRPC), a nationwide quality register [17, 18], the Cancer Survey [19], and Quality from the Patients’ Perspective Questionnaire (QPP) [20]. These questionnaires are all relevant for palliative care quality. Two of them, the Cancer Survey and the QPP is regularly used to evaluate care from the perspective of patients while SRPC uses a health care professional perspective. A total of 32 questions reflecting quality in terms of structure, process, and outcome were initially selected (SRPC: 23 questions (1-12, 25, 26, 31-34. 38), the Cancer Survey eight questions (20, 21, 28, 29, 30. 35-37), QPP: 1 question (13). Based on the fact that a majority of questions already used health care as responders the authors decided in concordance with the study aim to keep a health care perspective when developing the QPCQ-S. With such an approach the QPCO-S could also have the potential to serve as a base for future palliative care development. The questions and response options were modified to be relevant for palliative care of people with any diagnosis, any care setting, and at any time of illness. Questions and response options from the Cancer Survey and QPP were also modified to suite a healthcare professional perspective. For example, the question ‘Have you experienced not being taken seriously or being met with arrogance by healthcare staff?‘ was change to ‘At your place of work, how often do you perceive that patients are not taken seriously or are met with arrogance by healthcare staff?’.

Experts in palliative care and research; two nurses and one physiotherapist, all with a PhD degree, recruited from a palliative care research centre, received a request by mail to take part in an expert group and review the 32 questions suggested by the authors. They all accepted to participate and responded via e-mail. All questions were considered relevant, but some were thought to be indistinct and were therefore revised to improve readability, i.e., make them easier to understand and answer. Some relevant issues related to palliative care were perceived as not being covered; consequently, six new questions were added (15, 16, 19, 22, 23, 27), resulting in a preliminary version of the QPCQ-S consisting of 38 questions covering five areas: symptom management, conversations and support, participation, approach, and organisation and coordination of care. In addition, seven questions about background characteristics of the respondents, such as sex, age, profession, and experience of palliative care were included.

#### Check of relevance, coverage, and face validity

The preliminary version of the QPCQ-S was reviewed by an advisory group, separate from the expert group, that was recruited through head managers from different care contexts. This group included ten nurses in specialized palliative care, four nurse assistants from nursing homes, and one nurse and one social worker from oncology care. They were asked to provide written comments on overall coverage, as well as relevance and clarity of the questions. Based on their comments, the logical structure of questions, the response options, and relevance and clarity for each question were reconsidered by the authors. Finally, potential redundancy was considered, and the questions were standardised regarding tense.

To evaluate the response patterns of the preliminary version of the QPCQ-S, data were collected from 98 health care professionals at medical wards (*n* = 28) and nursing homes (*n* = 70). All participants had taken part in a development project with study circles aimed at improving palliative care. This project was initiated by a palliative centre responsible for palliative care development and research, and the participants received the questionnaire at place in the beginning of the study circles. The sample consisted of assistant nurses (*n* = 65), registered nurses (*n* = 22), physicians (*n* = 5), and paramedics (*n* = 6). The results showed some problems with floor and/or celling effect for several items. Therefore, items that had a four-point response scale that reflected a frequency (‘Always’, ‘Sometimes’, ‘Never’, ‘Don’t know’) were replaced with a more differentiated response scale (‘Always’. ‘Often’, ‘Sometimes’, ‘Rarely’, ‘Never’ or ‘Don’t know’).

#### Cognitive interviews

To evaluate understandability and clarity of the QPCQ-S, cognitive interviews with think-aloud protocols [21, 22] were conducted by three of the authors. Thirteen healthcare professionals in different care settings who treated patients with palliative care needs participated. They were recruited by a convenient approach based on professional contacts in primary home care (*n* = 2), nursing homes (*n* = 2), specialized palliative care (*n* = 3), surgical and medical wards (*n* = 6). The participants included registered nurses (*n* = 4), nurse assistants (*n* = 4), physicians (*n* = 3), paramedics (*n* = 1), and head of department (*n* = 1).

The respondents were asked to think aloud as they answered the questions. The interviews were audio recorded, and the interviewer made notes about verbal information as well as observations, such as if the respondent seemed to hesitate while answering a specific question. When completed, the interviewer asked retrospective probing questions to deepen the understanding about any problems that had occurred. For analysis, the notes and recordings were reviewed to identify problems with understanding, retrieval of information, estimation and answering the questions [23].

The analysis showed that participants had problems with one question concerning relief for seven different symptoms. As this question was considered complicated and difficult to understand, it was revised from ‘Does it happen that patients do not get the best possible symptom relief for the following symptoms’ to ‘How often do patients have symptoms that are not relieved’. Participants also commented on the lack of response options, for example, in a question about possibilities for overnight stay for next of kin, the response alternative ‘Sometimes’ was added. The final QPCQ-S is available as supplement to this study.

### Stage 2: overall evaluation of the QPCQ-S

An overall evaluation of QPCQ-S was performed as part of the baseline assessment of an educational intervention aiming to improve palliative care competence in nursing homes [16]. The sample included 421 participants with a mean age of 47.0 (SD = 10.5), a majority were female (*n* = 401, 95.3%). Different professions were represented; most were nurse assistants (*n* = 359, 85.3%) and registered nurses (*n* = 30, 7.1%) (Table 1). Quantitative data were presented with descriptive statistics. Regarding the three close-ended evaluation questions, Fishers exact test was used to compare differences between higher educated (registered nurses, occupational therapists, physio therapists, and head of departments) and lower educated (assistant nurses) healthcare professionals. The level of statistical significance was set at *p* < 0.05 and all analyses were conducted in Stata 18.0 for Mac (StataCorp, College Station, TX, USA). The participants answered the QPCQ-S and three close-ended evaluation questions: if the questions were important, difficult to answer, and provided them opportunity to give a true picture of palliative care at their workplace. In addition, the participants had the opportunity to answer an open-ended question considering comments on the questions and if they missed any question.

**Table 1:** Sample characteristics (*n* = 421)

Age, mean (SD) [range]	47.0 (10.5) [20–65]
Sex, n (%)	
Female	401 (95.3)
Male	18 (4.3)
Missing data	2 (0.5)
Health care profession, n (%)	
Registered nurse	30 (7.1)
Nursing assistant	359 (85.3)
Physio therapist	5 (1.2)
Occupational therapist	9 (2.1)
Head of department	9 (2.1)
Unknown	9 (2.1)
Years employed at current workplace, mean (SD) [range]	12.9 (9.6) [0–44]
Clinical experience of general palliative care, n (%)	
Yes	389 (92.4)
No	26 (6.2)
Missing data	6 (1.4)
Clinical experience of specialized palliative care, n (%)	
Yes	46 (10.9)
No	362 (86.0)
Missing data	13 (3.1)

The results are presented in Table 2. A great majority of the participants (*n* = 397, 99.3%) reported the questions to be important or fairly important in relation to quality of palliative care. Additionally, 263 (64.0%) participants reported the questions fairly difficult or difficult to answer, while 314 (78.9%) reported that the questions had provided them with the opportunity to give a true picture of palliative care.

**Table 2:** Evaluations of the questions in the QPCQ-S, by healthcare profession

	All HCP, *n* = 421	Professionals with academic education, *n* = 53	Nursing assistant, *n* = 359	Unknown profession, *n* = 9
The questions were, *n* (%)				
Important	296 (74.0)	36 (70.6)	252 (74.1)	8 (88.9)
Fairly important	101 (25.3)	15 (29.4)	85 (25.0)	1 (11.1)
Not that important	3 (0.8)	0 (0.0)	3 (0.9)	0 (0.0)
Not important at all	0 (0.0)	0 (0.0)	0 (0.0)	0 (0.0)
Missing data	21	2	19	0
The questions were, n (%)				
Easy	23 (5.6)	7 (13.2)	15 (4.3)	1 (11.1)
Fairly easy	125 (30.4)	16 (30.2)	107 (30.7)	2 (22.2)
Fairly difficult	208 (50.6)	27 (50.9)	175 (50.1)	6 (66.7)
Difficult	55 (13.4)	3 (5.7)	52 (14.9)	0 (0.0)
Missing data	10	0	10	0
The questions give a true picture of palliative care, n (%)				
Yes	314 (78.9)	34 (65.4)	275 (81.4)	5 (62.5)
No	84 (21.1)	18 (34.6)	63 (18.6)	3 (37.5)
Missing data	23	1	21	1

Professionals with academic education included registered nurses, occupational therapists, physio therapists, and head of departments

There was no significant difference between different healthcare professions regarding the importance of the items in the questionnaire (*p* = 0.670). However, nurse assistants rated the items as more difficult to answer compared to nurses, physicians or other professionals with academic education (*p* = 0.031). In addition, nurse assistants reported to a greater extent that the items gave a true picture of palliative care, compared to the other participants (81.4% vs. 65.4%, *p* = 0.016).

The open-ended question was answered by 116 (27.6%) participants. These comments revealed that the questions were difficult to answer for professionals other than registered nurses and physicians. *‟Some of the questions are not relevant for us*,* as care staff; nurses are better suited to answer them.”* This was related to the assignments that the professional had, how the work was organized and documented, and how communication took place between different healthcare professions. This was exemplified by quotes such as: *‟Unfortunately*,* as an assistant nurse*,* I lack knowledge about some routines and therefore I haven’t been able to answer the questions in a good way.”* and *“Don’t have full insight into written routines and how much nurses and doctors document.”*. Another quote stated *“Many questions that I don’t have the answers to! Became a buzzer concerning poor communication between doctor and nurse towards the assistant nurse.”.* Healthcare professionals working night shifts noted that the questionnaire was hard to answer since they felt they did not keep up with the organisation in the same way day shift professionals did. *“As I work the night shift*,* I’m not updated on the daily routines.”* Some experienced the questionnaire as extensive and containing lots of text, therefore taking a long time to complete. No suggestions on further questions appeared, which was interpreted as the questionnaire taking up relevant aspects of palliative care.

## Discussion

This study describes the development and evaluation of the QPCQ-S that aims to evaluate quality of palliative care from the perspective of healthcare professionals in different care settings. Overall, the questionnaire showed good convergent validity, and the questions were reported as important, giving a true picture of palliative care. However, the questions were reported as difficult to answer, particularly by the nurse assistants.

The findings showed that the questionnaire had good coverage, and that content validity was supported. In addition, a vast majority of the healthcare professionals included in the overall evaluation of the QPCQ-S reported that the items were important, independent of education level. These findings indicate that the instrument covers important aspects of palliative care and that it can therefore be used to identify potential areas for organizational improvements. For example, the QPCQ-S has been used to evaluate an educational intervention in nursing homes in a pre-post design, showing significant improvements concerning for example the use of valid instruments for symptom assessment [24].

Despite the fact that the questions were considered as important, they were found to be difficult to answer, particularly among nurse assistants working in nursing homes. One possible reason is that medical and care-related information is not always available and presented to all professionals, particularly nurse assistants. The fact that most participants were nurse assistants may therefore explain why the majority of the participants reported difficulties in answering some questions. It may also be an indication that some of this information needs to be shared in the whole team so that all members can participate in providing high quality palliative care. The results could also be a consequence related to the care context, for example nursing homes, where the professionals often lack specialist palliative care competence [25, 26]. Therefore, users of the QPCQ-S need to consider who is suitable to complete the instrument; however, it must also be remembered that when questions are experienced as difficult to complete for some health professional groups, it might reflect limitations in the palliative care provided. Perhaps, the QPCQ-S could form the base for care development and engage head managers and leaders. It can for example be used to share information about important local guidelines, inspire discussions about the palliative care approach and make all professionals feel involved in the work for best possible palliative care quality. Concerning future use, it should be noted that the QPCQ-S has been developed as a collection of questions that can be used together or individually, depending on relevance for the specific workplace or research project. Therefore, psychometric evaluations of unidimensionality and internal consistency is not relevant for the QPCQ-S.

Finally, some healthcare professionals found the questionnaire extensive. The reason for the large number of questions is that the aim was to develop a comprehensive tool covering different aspects of the quality of palliative care, including the most important indicators highlighted in national and international guidelines. However, there is a risk that the length may result in decreased completion rates and lower reliability of the responses [27]. Additionally, if only highly motivated individuals complete the questionnaire, the results may be biased. Therefore, it is important to point out that not all questions have to be used; the users of the QPCQ-S can select questions that are most important for the actual situation.

### Methodological considerations

The content validity index was not used in the present study, a method commonly recommended in instrument development [28]. Instead, to ensure content validity, an expert group participated in the process of constructing of the QPCQ-S. Further, the questions were developed from previous well-validated questionnaires, with documented content validity [17–20]. It should also be noted that cognitive interviews contribute with validity evidence regarding item content [29]. Cognitive interviews were also used to identify possible sources of response problems that were taken into consideration [21, 22]. In future research, evaluation of other aspects of measurement properties, such as stability and responsiveness, could be of importance.

Since the aim was to capture healthcare professionals’ perspective the consumers of palliative care were not involved in the development, something that can be considered a limitation. However, as this questionnaire is based on guidelines and quality indicators linked to how good palliative care should be carried out it can be argued that consumers of palliative care in some ways are present.

## Conclusion

The QPCQ-S was developed as a questionnaire to evaluate quality of palliative care in different care settings. The questionnaire showed good content validity in the present study, and the questions were reported to be important, giving a true picture of palliative care. However, the questions were reported as difficult to answer, particularly among nurse assistants, in nursing home context. Therefore, careful preparation is needed considering by whom, in what context and in what way the CPCQ-S is best used for evaluation of palliative care.

## Supplementary Information

Supplementary Material 1.

## Data Availability

The Quality of Palliative Care Questionnaire – Staff is available as supplementary material.

## Abbreviations

NBHW: The Swedish National Board of Health and Welfare
QPCQ-S: Quality of Palliative Care Questionnaire
SRPC: The Swedish Register of Palliative Care
