# Supplementary material for: Evaluating quality of palliative care from the perspective of healthcare professionals in different care settings: development of the quality of palliative care questionnaire – staff
Source: BMC Palliat Care. 2026 Apr 10;25:98. doi: 10.1186/s12904-026-02084-2 (PMC13077797; doi:10.1186/s12904-026-02084-2)
Supplement: Supplementary file 1 — Supplementary Material 1. [file 12904_2026_2084_MOESM1_ESM.pdf]

Quality of Palliative Care Questionnaire – Staff (QPCQ-S)

The following questions are about your perceptions of how palliative care is delivered at your workplace.

SYMPTOM RELIEF AND NURSING CARE

1) At your place of work, how often are patients' symptoms assessed using a validated scale, such as VAS?

- ❑ Always
- ❑ Often
- ❑ Sometimes
- ❑ Rarely
- ❑ Never
- ❑ Don't know

2) How often are the symptom assessments documented?

- ❑ Always
- ❑ Often
- ❑ Sometimes
- ❑ Rarely
- ❑ Never
- ❑ Don't know

3) How often do patients experience symptoms that are not relieved?

Symptoms:	**Never**	**Rarely**	**Sometimes**	**Often**	**Always**	**Don't know**
Pain	❑	❑	❑	❑	❑	❑
Death rattle	❑	❑	❑	❑	❑	❑
Nausea	❑	❑	❑	❑	❑	❑
Anxiety	❑	❑	❑	❑	❑	❑
Dyspnoea	❑	❑	❑	❑	❑	❑
Confusion	❑	❑	❑	❑	❑	❑
Depression	❑	❑	❑	❑	❑	❑

4) How often is there an individual prescription of PRN drugs in the medical record (for patients who may need it)?

Drugs against:	**Never**	**Rarely**	**Sometimes**	**Often**	**Always**	**Don't know**
Pain	❑	❑	❑	❑	❑	❑
Death rattle	❑	❑	❑	❑	❑	❑
Nausea	❑	❑	❑	❑	❑	❑
Anxiety	❑	❑	❑	❑	❑	❑
Dyspnoea	❑	❑	❑	❑	❑	❑
Confusion	❑	❑	❑	❑	❑	❑

5) How often is there access to injectable drugs?

Drugs against:	**Never**	**Rarely**	**Sometimes**	**Often**	**Always**	**Don't know**
Pain	❑	❑	❑	❑	❑	❑
Death rattle	❑	❑	❑	❑	❑	❑
Nausea	❑	❑	❑	❑	❑	❑
Anxiety	❑	❑	❑	❑	❑	❑
Dyspnoea	❑	❑	❑	❑	❑	❑
Confusion	❑	❑	❑	❑	❑	❑

6) When needed, how often are specialists outside the team/ward consulted?

❑ Always
❑ Often
❑ Sometimes
❑ Rarely
❑ Never
❑ Don't know

7) How often are fluids supplied by parenteral drip/feeding tube to patients during their last 24 hours of life?

- ❑ Never
- ❑ Rarely
- ❑ Sometimes
- ❑ Often
- ❑ Always
- ❑ Don’t know

8) How often is nutrition supplied by parenteral drip/feeding tube to patients during their last 24 hours of life?

- ❑ Never
- ❑ Rarely
- ❑ Sometimes
- ❑ Often
- ❑ Always
- ❑ Don’t know

9) How many of your patients suffer from pressure ulcers?

- ❑ None
- ❑ A few
- ❑ Less than 50%
- ❑ 50% or more
- ❑ Don’t know

10) How often are patients’ pressure ulcers documented?

- ❑ Always
- ❑ Often
- ❑ Sometimes
- ❑ Rarely
- ❑ Never
- ❑ Don’t know

11) How many of your patients receive regular assessments of their oral health?

- ❑ All
- ❑ Most
- ❑ 50%
- ❑ A few
- ❑ None
- ❑ Don't know

12) How often are assessments of patients' oral health documented?

- ❑ Always
- ❑ Often
- ❑ Sometimes
- ❑ Rarely
- ❑ Never
- ❑ Don't know

Comments about symptoms and nursing care:

____

____

____

____

____

ORGANISATION AND COORDINATION

13) How do you perceive that palliative care is carried out at your workplace?

- ❑ Completely based on the patients' needs
- ❑ Mostly based on the patients' needs
- ❑ Based equally on the patients' needs and healthcare routines
- ❑ Mostly based on hospital routines
- ❑ Completely based on hospital routines

14) Are there written guidelines, local or national, concerning:

	Yes	No	Don't know
How patients should be informed about transition to end-of-life care	❑	❑	❑
How next of kin should be informed about transition to end-of-life care for the patient	❑	❑	❑
How physicians should document transition to end-of-life care for the patient	❑	❑	❑
How support should be offered to the patients' next of kin	❑	❑	❑
How symptom assessments should be performed	❑	❑	❑
How common symptoms should be relieved	❑	❑	❑
What PNR drugs should be prescribed	❑	❑	❑
How staff should observe specific requests based on ethnical background	❑	❑	❑
How staff should observe spiritual/religious needs	❑	❑	❑
What actions should be taken when a death occurs	❑	❑	❑
How follow-up talks after a death should be offered to next of kin	❑	❑	❑

15) How many of your patients have a named healthcare contact?

- ❑ All
- ❑ Most
- ❑ 50%
- ❑ A few
- ❑ None
- ❑ Don't know

16) How many of your patients are cared for by a multi-professional team that cooperates regarding the patient's needs?

- ❑ All
- ❑ Most
- ❑ 50%
- ❑ A few
- ❑ None
- ❑ Don't know

17) On average, how long does it take when a patient needs access to immediate consultation during <u>office hours</u>?

By:	**Less than 2 hours**	**2-12 hours**	**More than 12 hours**	**Don't know**
Physician	❑	❑	❑	❑
Nurse	❑	❑	❑	❑
Assistant nurse	❑	❑	❑	❑
Social worker	❑	❑	❑	❑
Physiotherapist	❑	❑	❑	❑
Occupational therapist	❑	❑	❑	❑
Spiritual leader	❑	❑	❑	❑

18) On average, how long does it take when a patient needs access to immediate consultation during <u>on-call hours</u>?

By:	**Less than 2 hours**	**2-12 hours**	**More than 12 hours**	**Don't know**
Physician	❑	❑	❑	❑
Nurse	❑	❑	❑	❑
Assistant nurse	❑	❑	❑	❑
Social worker	❑	❑	❑	❑
Physiotherapist	❑	❑	❑	❑
Occupational therapist	❑	❑	❑	❑
Spiritual leader	❑	❑	❑	❑

Comments about organisation and coordination:

____

____

____

____

____

CONVERSATIONS AND SUPPORT

19) How many of your patients and their next of kin are asked if they want information/conversations separately or together?

- ❑ All
- ❑ Most
- ❑ 50%
- ❑ A few
- ❑ None
- ❑ Don’t know

20) Do you perceive that patients and their next of kin receive information in a way that makes it difficult to understand?

- ❑ Never
- ❑ Rarely
- ❑ Sometimes
- ❑ Often

21) How often are patients offered support regarding emotional and existential needs?

- ❑ Always
- ❑ Often
- ❑ Sometimes
- ❑ Rarely
- ❑ Never
- ❑ Don’t know

22) How often are the patient's next of kin offered support regarding emotional and existential needs?

- ❑ Always
- ❑ Often
- ❑ Sometimes
- ❑ Rarely
- ❑ Never
- ❑ Don't know

23) How often are the patient's next of kin asked about their need for support?

- ❑ Always
- ❑ Often
- ❑ Sometimes
- ❑ Rarely
- ❑ Never
- ❑ Don't know

24) How often is it possible for the patient's next of kin to:

	**Always**	**Sometimes**	**Never**	**Not applicable**
Sleep in the same room as the patient?	❑	❑	❑	❑
Sleep in another room?	❑	❑	❑	❑

25) Are next of kin offered a follow-up talk after the patient's death?

- ❑ Yes, always
- ❑ Yes, often
- ❑ Yes, sometimes
- ❑ No
- ❑ Don't know

26) How often are individually tailored and informed conversations about transition to end-of-life care carried out with patients?

- ❑ Always
- ❑ Often
- ❑ Sometimes
- ❑ Rarely
- ❑ Never
- ❑ Don't know

27) How often are individually tailored and informed conversations about transition to end-of-life care carried out with patient's next of kin?

- ❑ Always
- ❑ Often
- ❑ Sometimes
- ❑ Rarely
- ❑ Never
- ❑ Don't know

Comments about conversations and support:

____

____

____

____

____

PARTICIPATION

28) How often are the patient's next of kin asked if and how they want to participate in the care?

- ❑ Always
- ❑ Often
- ❑ Sometimes
- ❑ Rarely
- ❑ Never
- ❑ Don't know

29) Are next of kin given an opportunity to participate in decisions regarding the patient's care?

- ❑ Yes, always
- ❑ Yes, often
- ❑ Yes, sometimes
- ❑ No
- ❑ Don't know

30) Are patients given an opportunity to participate in decisions regarding their own care?

- ❑ Yes, always
- ❑ Yes, often
- ❑ Yes, sometimes
- ❑ No
- ❑ Don't know

31) How many of your patients are asked about their wishes concerning preferred place of death?

- ❑ All
- ❑ Most
- ❑ Some
- ❑ None
- ❑ Don't know

32) How often are the patients' wishes concerning preferred place of death documented?

- ❑ Always
- ❑ Often
- ❑ Sometimes
- ❑ Rarely
- ❑ Never
- ❑ Don't know

33) How often are the patients' expressed wishes concerning preferred place of death met?

- ❑ Always
- ❑ Often
- ❑ Sometimes
- ❑ Rarely
- ❑ Never
- ❑ Don't know

34) How often do patients die without having next of kin, staff, or another person present?

- ❑ Never
- ❑ Rarely
- ❑ Sometimes
- ❑ Often
- ❑ Always
- ❑ Don't know

Comments about participation:

____

____

____

____

____

ENCOUNTERS

35) How often do you perceive that patients are not taken seriously or are met with arrogance by staff at your workplace?

- ❑ Never
- ❑ Rarely
- ❑ Sometimes
- ❑ Often

36) How often do you perceive that patients are offended or badly treated by staff at your workplace?

- ❑ Never
- ❑ Rarely
- ❑ Sometimes
- ❑ Often

37) In general, how do you perceive that staff encounter patients with palliative care needs at your workplace?

- ❑ Very well
- ❑ Fairly well
- ❑ Fairly badly
- ❑ Very badly

Comments about encounters:

____

____

____

____

____

SATISFACTION WITH CARE

38) At your workplace, how satisfied are you with the care delivered to patients with palliative care needs?

**Not at all satisfied**				**Completely satisfied**
1	2	3	4	5
❑	❑	❑	❑	❑

Thank you for your participation!
